# Lower-Than-Expected Vitamin A Concentrations in a Commercial Diet Associated with Uroliths and Pyelonephritis in Rats

**DOI:** 10.3390/ani12101288

**Published:** 2022-05-18

**Authors:** John S. Munday, Fernanda Castillo-Alcala, Patricia Jaros

**Affiliations:** 1 School of Veterinary Science, Massey University, Palmerston North 4410, New Zealand; f.castillo-alcala@massey.ac.nz; 2 Invetus NZ, Ltd., Palmerston North 4410, New Zealand; pjaros@invetus.com

**Keywords:** vitamin A, toxicity study, nutrition, vitamin deficiency, experimental diets, nephrolith

## Abstract

**Simple Summary:**

Rats are often used to screen new foods or medications to ensure they are safe for human consumption. As the presence of lesions in a rat in such a study could indicate toxicity, it is essential that rats do not develop background lesions due to other diseases. In the present report, around 5% of rats in an oral safety study developed uroliths, with two of the affected rats also developing kidney infections. Histology was suggestive of vitamin A deficiency. Analysis of the food revealed much lower levels of vitamin A than stated on the label, but enough vitamin A to meet the published minimal nutritional requirements of rats. When the study was continued using rats fed food from a different source, no urinary tract lesions were observed. This suggests dietary factors may have caused the development of the uroliths in this case. It is hypothesized that the lower-than-expected concentration of vitamin A in the diet, potentially along with other dietary and environmental factors, resulted in squamous metaplasia of the uroepithelium and subsequent uroliths and pyelonephritis. The observations highlight the need to use high-quality diets for safety studies in rats.

**Abstract:**

Five of 95 rats in an oral safety study developed uroliths, with two of these rats also developing pyelonephritis. Histology of the urinary tract revealed squamous metaplasia suggestive of vitamin A deficiency. Analysis of the diet showed around half the expected concentration of vitamin A, although the concentrations were close to the published nutritional requirements for rats. Due to the presence of squamous metaplasia of the transitional epithelium and the low vitamin A concentration in the diet, a presumptive diagnosis of vitamin A deficiency was made, although an interaction between the low vitamin A concentrations and other dietary components appears possible. Although the uroliths did not cause clinical signs of disease, the lesions observed during this study could have been misinterpreted as being due to the test substance. Observations from this study highlight the need for high-quality food to ensure background lesions do not develop when performing safety studies in rats.

## 1. Introduction

Rats are commonly used to help ensure new food ingredients or medications are safe for use in humans or other animal species [1]. This is done by dosing the animals and then performing a necropsy to detect pathological changes. If changes are detected, this indicates that the test substance could cause toxicity at the dose that was tested. As something that causes lesions in rats could potentially also cause toxicity in other species, this can result in the test substance being altered to ensure safety or in the development of the new food ingredient or medication being abandoned. As identifying lesions in rats during safety testing can have significant ramifications for the development of the test substance, it is essential to use rats that are free of any other diseases. For this reason, rats used in safety studies are regularly tested to ensure they are specific-pathogen-free, the environment is tightly controlled, and rats are fed standardized diets that meet all nutritional requirements [2].

In the present case, female rats were used in a safety study to evaluate novel probiotic preparations. Uroliths were detected in around 5% of the rats, and two rats also developed pyelonephritis. Histology revealed squamous metaplasia of the urinary bladder. As uroliths and squamous metaplasia of the bladder have been previously reported in rats fed diets deficient in vitamin A [3,4], samples of the diet were analyzed. This revealed the diet contained lower concentrations of vitamin A than stated on the feed’s label, although the concentrations were above those currently recommended for growing rats [2,5]. There are previous reports of ‘outbreaks’ of urolithiasis in rats—one report each for Long–Evans [6] and Sprague–Dawley (SD) [4] rats—and the present report is the third report of multiple rats within a group developing uroliths. Evidence from these rats suggests that squamous metaplasia and uroliths can develop even when apparently adequate vitamin A is present in the diet. This may suggest an interaction between low levels of vitamin A in the diet and other environmental or dietary components in these rats. Observations from this case serve as a reminder that diets used for laboratory animals can be variable and highlight the importance of considering all possible factors when investigating unexpected lesions in safety studies.

## 2. Materials and Methods and Results

The study consisted of 95 female SD rats that were all sourced from the same breeder. The rats arrived at the animal facility at 5–7 weeks of age and were acclimatized for a further 5–6 weeks. At this time, 90 rats were provided via gavage with either 2 mL of a solution containing probiotic bacteria suspended in a solution consisting of 20% sucrose dissolved in phosphate-buffered saline (PBS) or a control solution that only contained sucrose dissolved in PBS. Five of the rats were not provided with any solution via gavage. During the acclimatization and treatment periods, the rats were housed in groups of three animals per cage in a climate-controlled room (19–24 °C, humidity ranging 35–70%) on a 12 h light/dark cycle. All rats had ad libitum access to both food and water. The rats were fed pelleted food from a commercial supplier (Feed A). The supplier was a small, regional company, although the food was stated to be consistent with National Research Council guidelines regarding the nutritional needs of rats. This diet was chosen as it was the same diet used by the breeder of the rats.

One of the rats was injured during the gavage procedure, necessitating euthanasia. In addition to traumatic injuries, necropsy of this rat revealed a 4 mm diameter pale, roughly spherical, slightly roughened urolith within the bladder.

The remaining 94 rats were monitored for two weeks after gavage, during which time they continued to gain weight as expected. Two weeks after gavage, the rats were euthanatized, and a necropsy was performed. During necropsy, five rats were observed to have a urolith present within the bladder. These uroliths ranged from 3 to 8 mm in diameter and were pale, roughly spherical, and had a mildly roughened surface. None of the rats had multiple uroliths within the bladder. As the uroliths were not sent to a laboratory for analysis, their composition is unknown. Examination of the kidneys of one of the affected rats revealed unilateral renal enlargement with the presence of pale streaks extending from the medulla to the cortex and a 2 mm urolith within the renal pelvis. Examination of both kidneys of another of the affected rats revealed the presence of multifocal-to-coalescing white nodules which were up to 4 mm in diameter. Dilation of the renal pelvis was also visible within both kidneys, although neither kidney contained a nephrolith. Samples of kidney and bladder from both rats with renal lesions and samples of bladder from another rat with a urolith were fixed in formalin and prepared for histological examination. Of the five rats with uroliths, four rats had received probiotic bacterial strains, while one rat had not been administered solution via gavage. Both rats with renal lesions had received probiotic bacteria.

**Figure 1:**
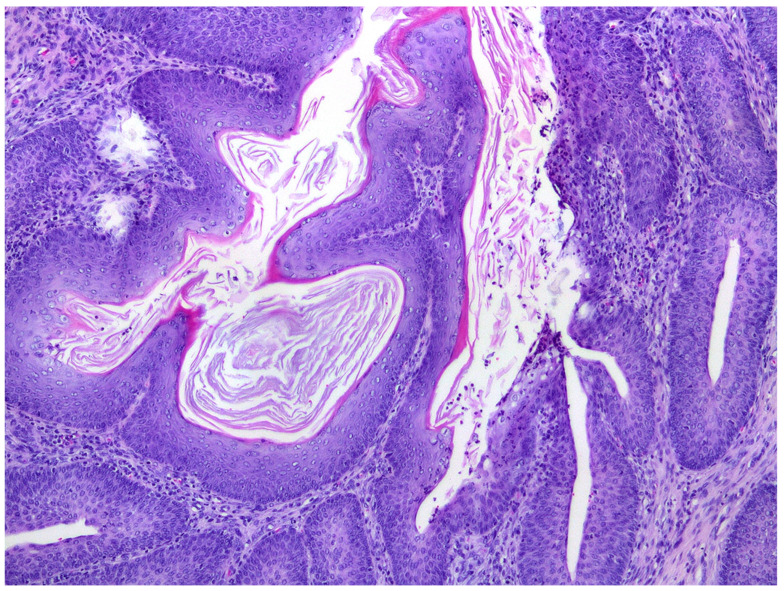
Photomicrograph of the bladder wall of a rat that had a urolith. The uroepithelium is thickened and demonstrates prominent squamous metaplasia. HE 200×.

**Figure 2:**
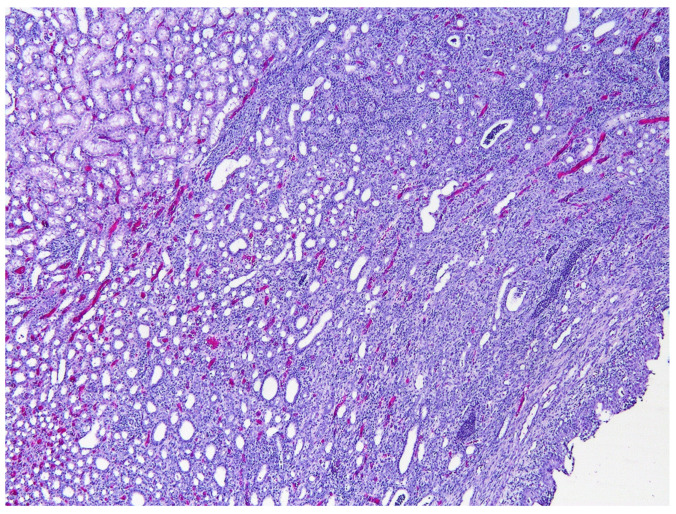
Photomicrograph of a kidney from the same rat as in Figure 1. The renal pelvis (lower right) is lined with thickened uroepithelium. The renal parenchyma has been infiltrated by large numbers of inflammatory cells, and inflammatory cells are visible within, and dilating, tubules. More normal renal parenchyma is visible within the top left of the figure. HE 50×.

Histological sections of the urinary bladder revealed mild to moderate urothelial hyperplasia characterized by irregular mucosal papillary projections lined by hyperplastic urothelium and focal areas of squamous metaplasia with moderate numbers of mixed inflammatory cells, including lymphocytes, plasma cells, and neutrophils (Figure 1). Neutrophils were commonly present migrating through the urothelium. Varying degrees of proprial oedema and inflammation were also present. Histology of sections of kidney revealed interstitial nephritis that affected up to 80% of the examined renal parenchyma and extended into the collecting ducts in the inner medulla and papilla. In additional, tubular inflammation, degeneration, and necrosis were visible within the areas of renal inflammation (Figure 2). Numerous tubular protein casts were present in some samples. Chronic changes were also present, including interstitial fibrosis and mild urothelial hyperplasia within the renal pelvis.

The squamous metaplasia of the urothelial epithelium was considered suggestive of vitamin A deficiency. Feed A was stated by the manufacturer to contain 3000 μg/kg of added vitamin A in addition to the vitamin A present in other food components. However, when three samples of this feed from different bags were analyzed by high-performance liquid chromatography [7], the measured concentrations of vitamin A were 1900 μg/kg, 1780 μg/kg, and 1220 μg/kg, respectively.

Due to the development of urinary tract lesions in the initial 95 rats, a further 300 female SD rats were obtained from a different supplier to complete the safety evaluation. These rats had been raised on Teklad Global 18% Protein Rodent Diet (Teklad Diets, Madison, WI, USA), and the rats continued to be fed this diet during the study. While this diet was not analyzed with regard to its vitamin A content, this diet was stated by the manufacturer to contain 4500 μg/kg of added vitamin A in addition to the vitamin A provided by other components of the diet. None of the 300 rats that were subsequently examined at necropsy had uroliths or any other grossly visible lesions in the urinary tract.

## 3. Discussion

Vitamin A deficiency was suspected in the present case due to the presence of squamous metaplasia of the transitional epithelium. Vitamin A regulates epithelial cell growth and differentiation, and squamous metaplasia of the urinary epithelium has been previously reported in rats fed diets containing <25% of the recommended amount of this vitamin [4,8]. Squamous metaplasia, as well as changes to the composition of urine, increases the likelihood of urolith formation, and deficiency of vitamin A is a well-recognized cause of uroliths in humans as well as within rats [3,9,10]. The squamous metaplasia, presence of uroliths, and potentially reduced immune function then increase the likelihood of an ascending bacterial infection of the urinary tract, as was seen in two of the rats with uroliths [11,12]. As uroliths can develop due to other dietary factors, it is possible the uroliths developed initially, and the presence of the uroliths subsequently resulted in squamous metaplasia. While this possibility was considered, the small size of the uroliths and the marked squamous metaplasia visible in some areas suggest this is unlikely. Unfortunately, as neither blood nor liver samples were collected from the rats with uroliths, analysis of this possibility was not feasible.

Also supporting a diagnosis of vitamin A deficiency was the detection of around half the expected concentration of vitamin A in Feed A. However, while lower than expected concentrations were detected, the minimal requirement for dietary vitamin A in growing rats has been reported to be 700 μg/kg [2,5], with AIN-93 growth diets containing 1200 μg/kg [13]. None of the tested bags had less than 1200 μg/kg of vitamin A, with the lowest concentration measured at 1220 μg/kg. However, no food from bags used earlier in the study was available for analysis. Considering the variability in the vitamin A concentrations of the three bags of food analyzed, it is possible that some of the bags used earlier in the study contained less vitamin A than the three analyzed bags. Overall, the analysis suggests that the diets fed to the rats in the present case had marginal levels of vitamin A. As the rats had been fed Feed A since being weaned, this would have prevented them for storing any vitamin A, predisposing them to developing hypovitaminosis A due to only a mild or transitory dietary deficiency. The marginal vitamin A concentration within Feed A is also consistent with only around 5% of the rats developing uroliths. If the rats had been fed a diet that contained significantly less vitamin A than required, evidence from previous studies suggests that most rats would have developed lesions, with many showing clinical signs of disease [4,8]. It should be noted that the diet’s vitamin A concentration was analyzed after the completion of the study and once samples were examined histologically. As vitamin A degrades over time, it is possible that the diets fed to the rats, particularly at the start of the study, could have contained greater quantities of vitamin A. However, due to the storage conditions of the food (in a cool, dark room), such changes in vitamin A concentration would be expected to be minimal.

Considering the lower-than-expected vitamin A concentration in Feed A, it is also possible that other irregularities were present in this diet. If so, it is possible that other imbalances, such as in dietary calcium, magnesium, and phosphate [14], could have resulted in the marginal vitamin A deficiency being more likely to cause urolith development. In addition, there is evidence that vitamin A requirements are increased by stressful environmental factors [15]. While the environment was well-controlled in the present study, the rats could have been stressed prior to arriving at the site of the safety study, or there could have been environmental stressors that remained undetected during the study that could have resulted in increased dietary demands for vitamin A in these rats.

While the current requirement for vitamin A in rats is 700 μg/kg [5], AIN-93 growth diets contain 1200 μg/kg [13], and the two diets used in the current study were formulated to contain 3000 μg/kg (Feed A) and 4500 μg/kg (Tekland Diets). This suggests that feed manufacturers have recognized that 1200 μg/kg may be inadequate in some situations. The observations from this group of rats suggest that dietary vitamin A concentrations around 1200 μg/kg can be associated with the development of urolith formation, albeit potentially in combination with other factors in the diet. While the identification of lower-than-expected levels of vitamin A in Feed A was unexpected, a recent study similarly observed variability in some nutrients when ‘standard’ diets from different suppliers were evaluated [16].

A role of the diet in causing the uroliths was supported by the absence of any uroliths in the 300 rats that received the food from the large multinational company that was formulated to contain higher concentrations of vitamin A. However, in addition to the later rats being fed a different diet, these rats were also from a different supplier. While all rats used were SD, some breeds of rats are predisposed to developing uroliths [6], and it remains possible that the initial 95 SD rats were genetically predisposed to developing uroliths.

In addition to its role in epithelial differentiation, vitamin A is also important in maintaining normal immune function [11]. When severe deficiency is induced by feeding rats a diet that contains no vitamin A, rats show respiratory tract or skin infections, weight loss, corneal keratinization, and salivary gland enlargement, with around 80% of rats dying within 15 weeks. In contrast, the observations from the present rats suggest that a less severe deficiency of vitamin A appears to affect the urinary system of rats more than the respiratory tract and skin. A predominance of urinary tract lesions had previously been reported in a group of rats that were fed a diet containing 210 μg/kg of vitamin A [4].

Although the suspected marginal deficiency in vitamin A in these rats did not cause clinical disease, the development of the uroliths and kidney lesions could have been highly significant for the safety study. For example, if all six affected rats had been in one treatment group, this would have suggested the lesions were due to the probiotic bacteria that were being tested. This could then have resulted in an expensive and time-consuming investigation of potential mechanisms by which the bacteria could have caused disease. In the present group, a role of the probiotics could be easily excluded, as two affected rats had either not been administered solution via gavage or had died immediately after dosing.

## 4. Conclusions

In conclusion, the observations from these rats suggest that squamous metaplasia of the urinary tract and urolith development can occur even when diets contain more than the minimal requirements for vitamin A. Whether the apparent increased susceptibility to deficiency in these rats was due to unrecognized environmental factors or other imbalances within the diet is uncertain. However, due to the potential for such lesions to disrupt safety studies, it is important to be aware of potential differences between commercial diets, and the use of reliable, high-quality diets is essential when performing such studies. Laboratory animal veterinarians and pathologists should be aware of signs of dietary deficiencies, as these can develop even when using commercially produced laboratory animal food.

## Data Availability

Not applicable.
